# Bibliometric Analysis of Research Trends and Prospective Directions of Lung Microbiome

**DOI:** 10.3390/pathogens13110996

**Published:** 2024-11-14

**Authors:** Chunjing Du, Yi Zhang, Hanwen Zhang, Hua Zhang, Jingyuan Liu, Ning Shen

**Affiliations:** 1 Department of Pulmonary and Critical Care Medicine, Peking University Third Hospital, Beijing 100191, China; 2 Center of Infectious Disease, Peking University Third Hospital, Beijing 100191, China; 3 Peking University Health Science Center, Peking University, Beijing 100191, China; 4 Research Center of Clinical Epidemiology, Peking University Third Hospital, Beijing 100191, China; 5 Department of Critical Care Medicine, Beijing Ditan Hospital, Capital Medical University, Beijing 100015, China

**Keywords:** microbiome, lung, bibliometrics, CiteSpace, visualization

## Abstract

The lung microbiome has emerged as a pivotal area of research in human health. Despite the increasing number of publications, there is a lack of research that comprehensively and objectively presents the current status of lung microbiome-related studies. Thus, this study aims to address this gap by examining over two decades of publications through bibliometric analysis. The original bibliographic data of this study were obtained from the Web of Science Core Collection, focusing on publications from 2003 to 2023. The analysis included the data extraction and examination of authors, affiliations, countries, institutions, abstracts, keywords, references, publication dates, journals, citations, H-indexes, and journal impact factors. A total of 845 publications were identified, showing an increasing trend in both publications and citations over the years, particularly in the last decade. The analysis highlighted the most productive authors, institutions, and countries/regions, and identified potential partners for interested researchers. Co-citation analysis revealed that lung microbiome- and infectious/pulmonary disease-related studies are at the forefront of the field. The hotspots and frontiers of the lung microbiome field have progressed from basic composition to exploring specific mechanisms and the clinical value of diseases. In conclusion, this study provides a comprehensive overview of the current research status and trends in the field of the lung microbiome over the past two decades and highlights the areas that need more attention and research efforts. It offers valuable insights for researchers and institutions and identifies key hotspots and frontiers, which can serve as references for related researchers and future research.

## 1. Introduction

The healthy human lung, once considered a sterile environment, is now recognized as a dynamic ecosystem known as the lung microbiome [1,2,3]. This complex community of microorganisms, consisting of bacteria, viruses, fungi, and archaea, inhabits the respiratory tract and has garnered increasing attention in recent years [3]. Over the past two decades, next-generation sequencing and other molecular biology techniques have significantly advanced our insights into the lung microbiome’s composition and role, with implications for clinical diagnosis, treatment, and disease prevention [4,5]. Understanding the lung microbiome’s impact on health and disease is crucial for advancing diagnosis and developing innovative therapies. 

Current research indicates that the composition and diversity of the lung microbiome are related to different kinds of diseases, including but not limited to asthma, chronic obstructive pulmonary disease (COPD), cystic fibrosis, and lung cancer [6,7,8]. Numerous studies have demonstrated that alterations in the lung microbiome can influence disease susceptibility, progression, and treatment outcomes [1,5]. For instance, dysbiosis, or microbial imbalance, has been linked to increased inflammation and the exacerbation of respiratory diseases [9,10,11]. Interactions between lung and gut microbiota may also impact systemic inflammation and overall health [12,13]. For patients with COVID-19, modulating the lung and gut microbiota is emerging as a promising adjunctive approach for prevention or treatment [14]. Despite recent advances in the lung microbiome, the field still faces challenges and further research is warranted. 

Bibliometrics is a branch of informatics that focuses on the systematic quantitative and qualitative analysis of literature. This method can quantitatively measure research field contours, relationships, and clustering [15], establishing it as a key technique for assessing academic work quality, credibility, and impact [16,17]. Specifically, bibliometric analysis can assess the contributions and influence of authors, countries, institutions, and journals, as well as provide insights into the current state, trends, and frontiers of research activities [18,19,20]. This approach is particularly valuable in the context of rapidly evolving fields like lung microbiome research, where tracking progress and identifying knowledge gaps would inform future studies. 

Although the number of annual publications continues to grow rapidly, to our knowledge, there is a lack of comprehensive and objective studies related to the lung microbiome, including bibliometric analysis. To fill this knowledge gap, this study aims to carry out a bibliometric analysis of the publications related to the lung microbiome and systematically reveal the results of the research hotspots and trends over the past 20 years, providing a new perspective for future research in the lung microbiome field.

## 2. Methods

### 2.1. Data Collection

The original bibliographic data for the metrological analysis in this study were obtained from the Web of Science Core Collection (WOSCC), a comprehensive and standardized database widely utilized in academia [21]. Guided by Medical Subject Headings (MeSHs), the search strategy was formulated as: TI = (((lung) OR (respiratory)) AND ((microbiome) OR (microbiota))). The initial search yielded 2548 articles, covering the period from 1 January 2003 to 29 January 2024. Only articles written in English were considered for inclusion, resulting in a total of 845 articles without duplicates being incorporated into the subsequent bibliometric analysis. To reduce deviation and enhance credibility, two researchers (YZ and HZ) screened the literature independently. Any disagreement was resolved by discussion or consultation by the third author (CD).

### 2.2. Data Extraction

The extracted data from all the included studies comprised authors, affiliations, countries, institutions, abstracts, keywords, references, publication dates, journals, the total citations of all databases, Hirsch indexes (H-indexes), and journal impact factors (IFs). The countries and institutions were counted based on the corresponding authors. Journal IFs were obtained from Journal Citation Reports (JCRs) 2022 to reflect the academic influence. The author and H-index information including the country, institution, H-index, and research area was sourced from the Scopus database and Google Scholar. 

### 2.3. Data Analysis and Visualization

Bibliometric visualization was completed by CiteSpace (version 6.21. R4), including the journals, authors, countries/regions, affiliations, and keywords, as well as co-occurrence analysis and the clustering of keywords and co-citation networks. In addition, R software (version 4.3.1) was used for chart plotting and data statistics, as well as Microsoft (version 2016) for organizing the publications and analyzing their basic characteristics. The “pheatmap” package was used to plot the keyword correlation heatmap, and the data in the heatmap are all standardized results. The correlation between keywords displayed in the heatmap is consistent with the node correlation in the CiteSpace co-occurrence graph, using the node correlation formula described in Chen Chaomei’s book “Citespace: Scientific Text Mining and Visualization” [22]. The “ComplexHeatmap” package and “circlize” package of the R software were used to plot circular heatmaps that show keywords for different years. The Latent Semantic Indexing algorithm was used to extract labels for cluster labeling, including keyword clustering and co-citation clustering.

## 3. Results

### 3.1. Annual Publications and Citation Trends

**Figure 1:**
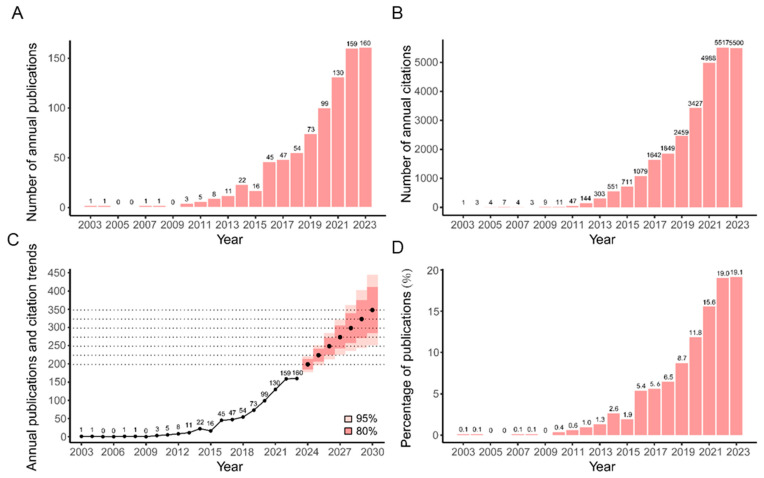
Publication and citation analysis. (**A**) Annual publications from 2003 to 2023. (**B**) Annual citations from 2003 to 2023. (**C**) Proportional distribution of publication volumes from 2003 to 2023. (**D**) Annual publication forecasts until 2030.

The characteristics of 845 publications related to the lung microbiome are demonstrated in Figure 1. According to the citation report of Web of Science (WOS), these publications have been cited a cumulative total of 13,633 times, averaging 34.67 citations per year. As shown in Figure 1A–C, the number of annual publications and citations was relatively low in the initial decade (2003–2012), with no more than 10 publication outputs and 150 citations per year. However, since 2013, there has been a noticeable upward trend, with annual publications surpassing 50 in 2018 (*n* = 54, accounting for 6.4% of 845) and reaching over 100 in 2021 (*n* = 130, accounting for 15.4% of 845), and annual citations surpassing 1000 in 2016 (*n* = 1079, accounting for 7.9% of 13,633) and reaching over 5000 in 2022 (*n* = 5517, accounting for 40.5% of 13,633). Figure 1D presents the general prediction for the number of published articles from 2024 to 2030. The forecast shows a continued upward trend, with the annual number of publications potentially reaching 350 by 2030. 

### 3.2. Analysis of Published Journals

The retrieved publications in the present study were published in 557 journals, and the journals with more than 10 published articles are presented in Table 1. Among the 14 productive journals, the total number of publications with IFs above five was 185, accounting for 21.9%. Specifically, high-impact journals including the *American Journal of Respiratory and Critical Care Medicine* (*n* = 27), *Nature Communications* (*n* = 10), and *Microbiome* (*n* = 26), with IFs above 10, accounted for 7.5%, while journals like *Thorax* (*n* = 10), *Frontiers in Immunology* (*n* = 13), *Respiratory Research* (*n* = 20), *Frontiers in Cellular and Infection Microbiology* (*n* = 39), and *Frontiers in Microbiology* (*n* = 40), with IFs ranging from 5 to 10, represented 14.4%. Together, these journals comprise over 20% of the publications. Centrality represents the degree centrality of a journal, measuring the relationship between a journal and other journals in the co-citation network; high centrality means that a key node has a strong influence on the relationships in the network. Nodes with more than 0.1 mediation centrality become the key points. As shown in Table 1, there are no journals whose centrality is greater than or equal to 0.10 among all the journals. The journal co-citation network comprising 136 journals and 124 links is shown in Figure S1. Notably, high-IF journals such as *Lancet*, *Science*, the *American Journal of Respiratory and Critical Care Medicine*, and *Microbiome* serve as central nodes, exerting significant influence and occupying an important position in the co-citation network of journals in the lung microbiome field of research. 

### 3.3. Cooperative Relationship Network

#### 3.3.1. Country and Institutional Cooperation Network

**Figure 2:**
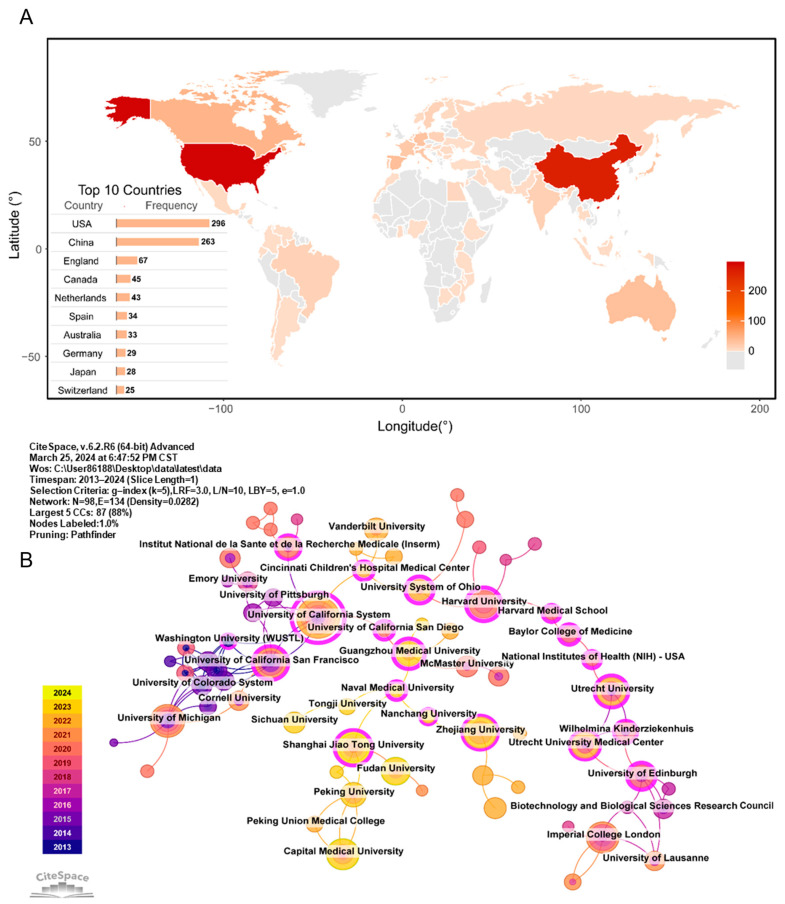
Analysis of countries and institutions. (**A**) Distribution of publication quantities worldwide. The table shows the top ten countries by publication count. (**B**) Collaborative network of publishing institutions.

Through statistical analysis of the number of published papers by countries/regions and institutions, we can identify the key contributors and their collaborative networks within the field. From 2003 to 2023, a total of 98 institutions in 71 countries/regions contributed to publications related to the lung microbiome. As shown in Figure 2A, the United States was the most prolific country in the field, with 296 publications, followed by China (*n* = 263), the United Kingdom (*n* = 67), Canada (*n* = 45), and New Zealand (*n* = 43). 

By utilizing CiteSpace software, the cooperative relationships between institutions and countries/regions were analyzed (Figure 2B and Figure S2). Each node represents a country or institution, and the color of the dot represents the year of publication. The larger the node, the higher the number of published papers, and the more yellow the node, the more recent it is. The line between the two nodes indicates the strength of the cooperative relationship between the two. The purple circle around the node indicates that the node is the central node, which has high centrality and greater influence in cooperation. In the institutional collaboration network (Figure 2B), there were three tightly knit groups: two in the Euro-American sphere, led by the University of California system and Harvard University, and one in China, anchored by Guangzhou Medical University. The University of California system served as a pivotal node bridging these three groups. The top institutions of publishers were predominantly in Europe and the United States, with Imperial College London taking the lead (*n* = 13), followed by McMaster University (*n* = 8, Table S1). As shown in Figure S2, the international cooperation network was primarily segmented into two clusters: one centered around Western countries, with the United States as a focal point, and another involving Asian countries, centered on China, along with developing nations. There was limited interaction between these two networks.

#### 3.3.2. Author Cooperation Network

**Figure 3:**
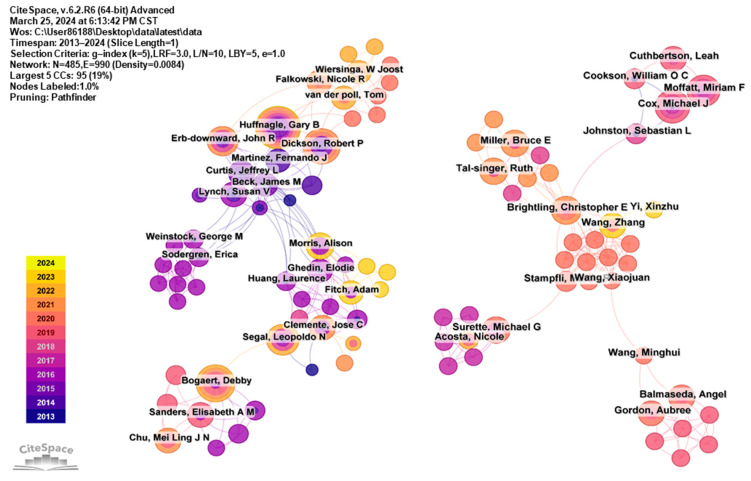
Collaborative author network.

By analyzing publication counts and collaboration networks, we identified 485 researchers contributing to the lung microbiome field, with 6 having authored over 10 articles on lung microecology (Table 2). Leading the list is Huffnagle, Gary B. from the University of Michigan, ranked first with 21 articles, followed by Bogaert, Debby with 15 articles and Dickson, Robert P., who has published 14. As shown in Figure 3, the cooperative relationships between scholars are distributed in a decentralized manner. We analyzed the citation clustering network of Huffnagle, Gary B., Bogaert, Debby, and Dickson, Robert P., the three most prolific authors in the field, to highlight the focus areas in lung microbiome research. As shown in Figure S3, the articles published by these three authors were concentrated in the last five years, mainly since 2019, with an emphasis on the relationship between the lung microbiome and pulmonary diseases.

### 3.4. Analysis of Hotspot Evolution

#### 3.4.1. Keyword Analysis

**Figure 4:**
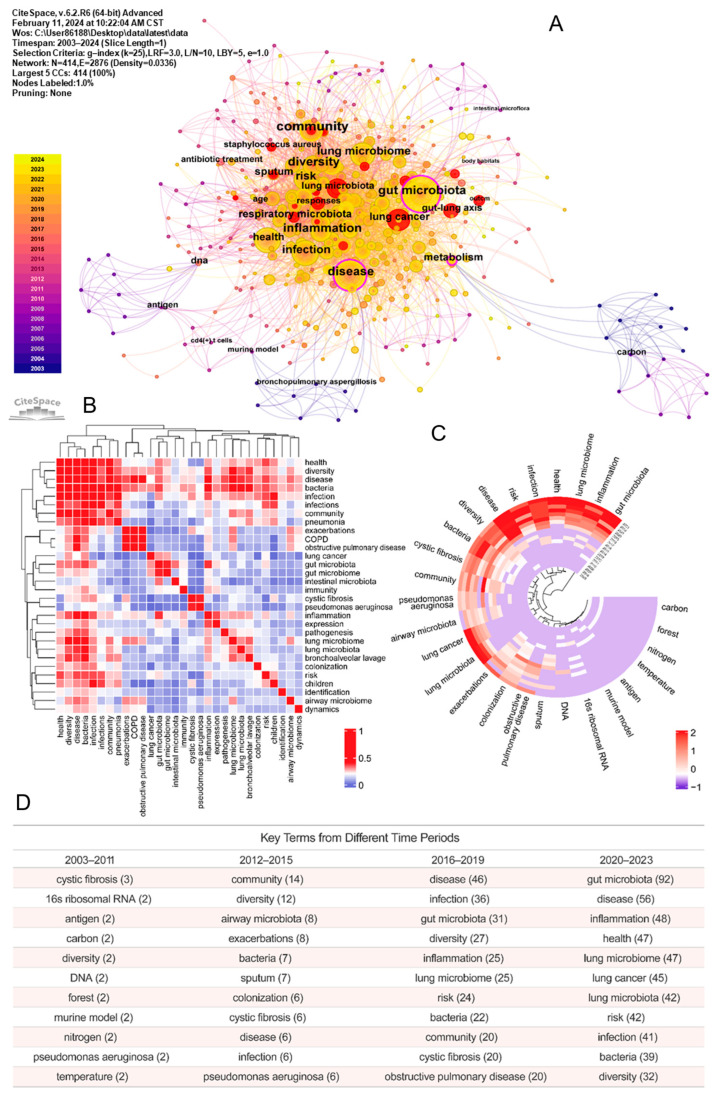
Keyword analysis. (**A**) Keyword co-occurrence network. (**B**) Heatmap of the top 30 keywords by occurrence correlations. The correlation between two keywords is calculated using the formula ‘c/(s1 * s2) ^ 0.5’ (s1 and s2 are the counts of documents containing each keyword in the title or abstract; c is the count of documents containing both keywords). The data in the figure have been standardized. (**C**) Changes in the number of important keywords over the years. The data in the figure have been standardized. (**D**) Statistics of important keywords by year.

**Figure 5:**
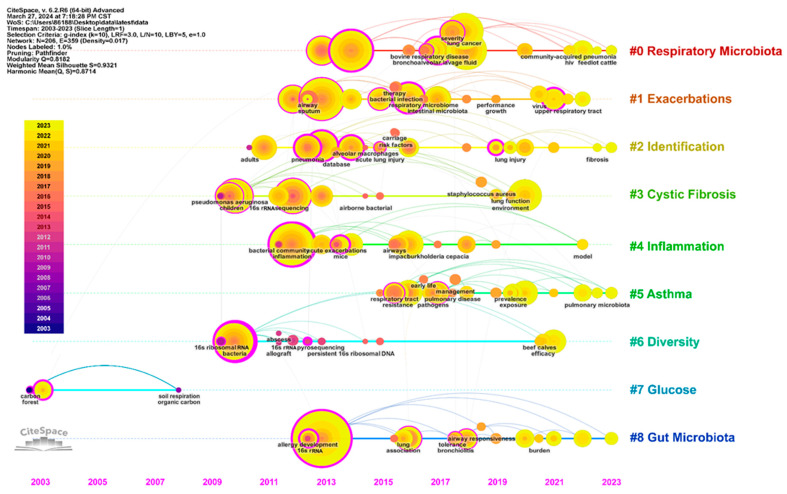
Evolution of keywords within each cluster over the years.

Using CiteSpace to analyze keyword co-occurrence, clustering, and burst is helpful to understand the research hotspots, frontiers, and trends in this field. As shown in Figure 4A, the number of keyword co-occurrence network nodes is 414 and the number of links is 2876. The top five high-frequency keywords are “gut microbiota” (*n* = 35), “disease” (*n* = 35), “infection” (*n* = 34), “inflammation” (*n* = 32), and “lung microbiome” (*n* = 32). The related keyword co-occurrence clusters are: #0 respiratory microbiota, #1 exacerbations, #2 identification, #3 cystic fibrosis, and #4 inflammation (Figure S4). As shown in Figure 4B, there is a close relationship between human health and the microbial community. Infection, diseases, COPD, and lung cancer are positively correlated with lung microbiome. The keywords are roughly divided into four stages (Figure 4C,D and Figure 5): from 2003 to 2011, different types of microorganisms were discovered through some sequencing techniques; from 2012 to 2015, the disease was found to be related to microorganisms; from 2016 to 2019, the interaction of the lung microbiome between immunity and gut microbiota was gradually brought into focus; and from 2019 to date, the in-depth research on the role of the lung microbiome in health and various diseases has gradually received increasing attention.

#### 3.4.2. Burst Detection

A citation burst means that the number of citations increases in a short period of time, so burst detection can reveal the most active areas and emerging trends in the network. Using the keyword outburst function of CiteSpace, the top 20 outburst keywords are shown in Table 3. The keywords with strong outbreak intensity in the last five years were “staphylococcus aureus” (2019–2020, strength: 3.37), “lung microbiota” (2019–2023, strength: 4.76), “injury” (2021–2023, strength: 3.91), “virus” (2021–2023, strength: 3.7), and “efficacy” (2021–2023, strength: 3.13). The ring heatmap and time axis view of keywords in Figure 4C and Figure 5 reveal a discernible trend in research, shifting from a global perspective—gaining a broad understanding of the microbiome—to a more localized focus, where attention is concentrated on specific mechanisms and the role of the microbiome in particular diseases or symptom clusters.

#### 3.4.3. Co-Citation Analysis

**Figure 6:**
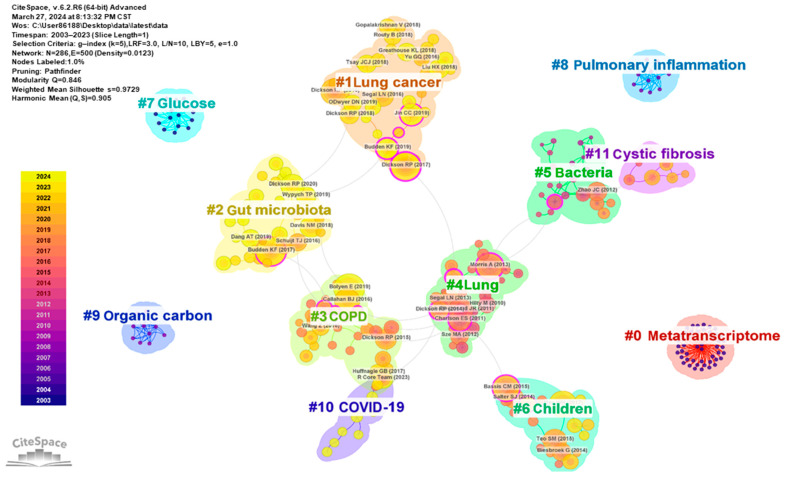
Co-citation keyword clustering.

Co-citation analysis identifies a relationship between two articles that are cited together, indicating a shared research foundation and a significant source of knowledge. The frequency of co-citation signifies an article’s importance in its field. A total of 641 citations from 2003 to 2024 were extracted to conduct a co-citation analysis (Figure 6 and Figure S5). The co-cited network contains 12 clusters with significant modularity and profile scores (Q = 0.846; S = 0.973). The top five clusters include #0 metatranscriptome, #1 lung cancer, #2 gut microbiota, #3 COPD, and #4 lung, all representing the recent literature. The top 30 most cited articles, with a minimum of 25 co-citations each, are predominantly post-2014 publications (Table S2). Double graph overlay is a method to analyze the relationship between existing research and future research trends. The double-graph superposition of the journal clustering is presented in Figure S6. The left side is the citing journals, and the right side is the cited journals. The labels indicate the disciplines covered by the journals, and colored paths indicate the citation relationships. In Figure S6, there are two important paths: the yellow citation path shows that research in molecular, biology, and immunology journals is frequently cited by molecular, biology, and genetics journals; the green citation path indicates that the research in medicine, medical, and clinical journals is frequently cited by molecular, biology, and genetics journals. These findings reveal that research on the lung microbiome has progressed from basic composition to exploring specific mechanisms and the clinical value of diseases.

## 4. Discussion

### 4.1. General Information

In this study, we systematically collected the bibliographic data on the lung microbiome from the core database of Web of Science and performed a comprehensive set of bibliometric analyses. We aimed to explore the intellectual structure, developing trajectory, and emerging trends in the lung microbiome field. Overall, we identified 845 English articles, cumulatively cited 13,633 times, published between 2003 and 2024. Although there were few articles at the beginning, the annual number of publications presented a rapidly increasing trend, especially in recent years. The rising number of publications, along with the increasing citation rates of both current and anticipated future works, suggests that the lung microbiome is a burgeoning field that has captured the interest of the research community and is garnering heightened attention. 

Based on the statistical analysis of the number of papers published by 98 institutions across 71 countries/regions, we can identify key countries/regions and research institutions that have significantly contributed to the field of the lung microbiome and assess their cooperative relationships. As shown in Figure 2, the United States, China, and the United Kingdom stand out as the primary hubs for lung microbiome research. Among the top ten institutions, most are in European and American countries. In these regions, robust collaborations are observed between the University of California system, Cornell University, the University of Michigan, and others, including Ohio University, Harvard University, and Utrecht University. In Asia, Guangzhou Medical University, Shanghai Jiao Tong University, and Zhejiang University exhibit strong connections, with Guangzhou Medical University also engaging in collaborations beyond the region. Notably, while European and American countries initiated their research on lung microecology earlier, Asian nations have progressively emerged and gained prominence in recent years. Enhanced cooperation and communication between countries and institutions are conducive to eliminating academic barriers and promote further advancements in lung microbiome research.

The H-index is a comprehensive quantitative index for evaluating researchers’ academic output quantity and level [23]. Among 485 researchers, Huffnagle, Gary B. from the University of Michigan Medical School in the U.S. leads with the highest H-index, highlighting his significant influence in the field of the lung microbiome. His work suggests that the lung microbiome not only affects disease susceptibility and causation but is also influenced by disease activity and response to treatment [3,24,25,26]. A deeper comprehension of these dynamics could lead to the development of novel therapies and preventive strategies, aiding in the identification of at-risk populations and improving targeted interventions [26,27]. This is followed by Bogaert, Debby from MRC Centre for Inflammation Research, United Kingdom. Bogaert, Debby focuses on the relationship between the respiratory microbiome and diseases in children and infants [28,29,30]. Using a multi-omic approach, Bogaert has revealed species-specific host–microbe interactions that could predict susceptibility to respiratory tract infections, contributing valuable insights to the field [31]. As is shown in Table 3, there are no authors whose centrality is greater than or equal to 0.10 among all the authors, indicating that there are no authors with significant influence in the lung microbiome field.

### 4.2. Hotspots and Frontiers

High-frequency keyword analysis helps us to understand the field frontiers and hotspots of the lung microbiome, and provides follow-up research directions for researchers [32]. The co-occurrence network diagram of high-frequency keyword analysis suggests several popular research directions, including INFECTION (pneumonia, pathogenesis, bacteria, pseudomonas aeruginosa, infection(s), etc.), PULMONARY DISEASE (obstructive pulmonary disease, COPD, lung cancer, cystic fibrosis, etc.), the LUNG–GUT AXIS (gut microbiota, gut microbiome, intestinal microbiome, lung microbiome, lung microbiota, airway microbiome, etc.), and IMMUNOLOGY (immunity, inflammation, etc.). Correspondingly, the timeline diagram analysis shows that respiratory microbiota, inflammation, and gut microbiota are all larger clusters, indicating that they have higher heat. Notably, keyword and co-cited literature analysis suggests that while research on “pneumonia”, “biodiversity”, and “inflammation exacerbation” is experiencing a slowdown in progress, detailed studies on “lung cancer”, “COPD”, and the “lung-gut axis” are gaining traction. Meanwhile, the double-graph superposition of the journal clustering indicates that research on the lung microbiome has progressed from basic composition to exploring specific mechanisms and the clinical value of diseases.

### 4.3. Lung Microbiome and Diseases

The link between the lung microbiome and disease has received increasing attention. Research on lung microbiome-related diseases focused on a series of diseases, including cystic fibrosis, asthma, infectious diseases, lung cancer, COPD, etc. “Lung Microbiome in Cystic Fibrosis” published by Filippo Scialo et al. elaborated the role of microbial–host interactions in the development of cystic fibrosis [33]. Similarly, the lung microbiome is crucial in the development of asthma [10,34], with infant cohort and animal studies revealing microbial signatures linked to asthma risk [35]. Furthermore, the lung microbiome is pivotal in infectious diseases, particularly pneumonia. The interaction between the lung microbiome and pneumonia is intricate, dynamic, and bidirectional. Early intensive care studies have demonstrated that probiotics can lower pneumonia risk and reduce the duration of mechanical ventilation in ICU patients [36]. Conversely, pneumonia’s development and progression can alter the microbiome’s composition and homeostasis [37,38]. With the outbreak of COVID-19 in 2020, studies linking the lung microbiome to severe acute respiratory syndrome coronavirus (SARS-CoV-2) have emerged as a key focus. Research indicates that a higher pulmonary microbial load in COVID-19 patients correlates with a reduced probability of recovery from invasive mechanical ventilation and increased mortality rates [39,40]. This may involve alveolar proinflammatory cytokines and altered pulmonary microbiota affecting the immune response and inflammation [41,42]. In addition, recent research has highlighted the lung microbiome’s role in lung diseases, particularly lung cancer and COPD [9,43]. Changes in the lung microbiome are correlated with lung cancer incidence, development, and prognosis, with cancer patients showing higher abundances of Streptococci and Staphylococci compared to non-cancerous individuals [11]. The dysregulation of the lung microbiome or the destruction of the gut–lung axis can cause DNA damage, genomic instability, and increased susceptibility to carcinogenic damage, contributing to lung cancer development [44,45]. In COPD, the lung microbiome’s diversity and microbial abundance differ significantly from those in healthy individuals, with these differences intensifying during exacerbations [46,47]. Changes in the dynamics of the lung bacterial microbiome could be attributable to disease heterogeneity, physiological changes, therapeutic interventions (e.g., antibiotics and corticosteroids), and exacerbations [48,49]. Investigating the lung microbiome’s functional role in COPD, beyond its contribution to exacerbations, remains an active area of research. 

The disorder of the lung microbiome has been linked to various diseases, making its detection as a disease biomarker and its targeted regulation for therapeutic purposes a burgeoning area of interest [9]. In patients with asthma and COPD, pathogenic Proteobacteria, especially Haemophilus, were increased, while in cystic fibrosis, Candida albicans was increased [50,51,52]. These findings indicate that the composition and diversity of the lung microbiome affects disease incidence, progression, and prognosis, suggesting its potential role as a diagnostic biomarker [5,53]. In addition, pulmonary microorganisms and their byproducts significantly affect clinical treatments, particularly immunotherapy. Both immunotherapy and prognostic outcomes decreased with altered microbial abundance [54]. Integrating microbial therapy into treatment may enhance treatment efficacy. For patients with COVID-19, modulating the gut and lung microbiota is emerging as a promising adjunctive approach for prevention or treatment, leveraging the immunomodulatory properties associated with probiotics and prebiotics [55]. Despite the current advancements in treatment approaches, the development of drugs designed to restore the lung microbiome balance is primarily focused on fundamental research. Further clinical trials are essential to validate their clinical benefits. In the future, developing specific and effective drugs to treat lung microbiome-related diseases will still be a research hotspot in this field.

### 4.4. Strengths and Limitations 

Compared to traditional extensive literature reviews, the CiteSpace- and R software-based bibliometric analysis offers a comprehensive and objective approach to data analysis. Additionally, we present results to explore relationships between the lung microbiome and related diseases, enhancing our understanding of research focuses and future trends in this field. Despite adherence to rigorous bibliometric principles, the study has certain limitations. First, only articles published within a specific period of time in the WOSCC were included, excluding non-SCI journals or other databases, which might introduce publication bias. Given WOSCC’s authority, the extended timespan of our search, and the significant overlap of articles in different databases, we believe our findings can reflect the current research trends and future hotspots in the lung microbiome. Second, the restriction to the English-language literature could introduce bias, potentially overlooking insights from non-English sources. Third, bibliometrics cannot assess the quality of individual studies, as citation metrics are influenced by the publication date, meaning more recent articles may be less cited than older ones primarily due to their publication date [56]. While these limitations might slightly impact the overall results, they are unlikely to alter the primary trends identified in this paper. 

## 5. Conclusions

This study provides an overview of the major research hotspots and frontiers in the field of the lung microbiome through data visualization. Specifically, the number of publications in this area has grown rapidly over the past decade. The United States leads in the quantity of publications, followed by China and the United Kingdom. Strengthening international collaboration, particularly with emerging nations, is essential to advance this field. The clinical and mechanism research on the lung microbiome in pulmonary diseases is currently a hotspot in this field. Despite substantial evidence suggesting that modulating the lung microbiome could be therapeutic for pulmonary diseases, clinical trials in this domain remain scarce. Thus, there is a critical need for more in-depth investigations into the clinical efficacy and safety of interventions targeting the lung microbiome. In conclusion, the findings of this study provide a foundation for understanding research topics, hotspots, and future directions in the lung microbiome, potentially illuminating pathways for enhanced diagnosis and therapeutic strategies for related diseases.

## Figures and Tables

**Table 1:** Journals with more than 10 publications.

Journal	2022 JIF	Record Count	Centrality
*Nature Communications*	16.6	10	0.01
*Thorax*	10	10	0.01
*Frontiers in Oncology*	4.7	11	0.01
*Frontiers in Immunology*	7.3	13	0.03
*Microbiology Spectrum*	3.7	14	0.01
*BMC Microbiology*	4.2	15	0.03
*Microorganisms*	4.5	17	0.00
*Respiratory Research*	5.8	20	0.02
*Microbiome*	15.5	26	0.01
*American Journal of Respiratory and Critical Care Medicine*	24.7	27	0.02
*Scientific Reports*	4.6	36	0.01
*Frontiers in Cellular and Infection Microbiology*	5.7	39	0.01
*Frontiers in Microbiology*	5.2	40	0.03
*PLoS ONE*	3.7	55	0.01

**Table 2:** Top 15 authors by publication count.

Author	Country	Institution	H-Index	Reasearch Area	Record Count	Centrality
Huffnagle, Gary B.	United States	University of Michigan Medical School	85	Immunology; Microbiology; Allergy	21	0.03
Bogaert, Debby	United Kingdom	MRC Centre for Inflammation Research	52	Pediatrics; Infectious Diseases; Microbiome; Respiratory	15	0.01
Dickson, Robert P.	United States	University of Michigan Medical School	41	Pulmonary Medicine; Microbiology; Pneumonia; ARDS; Critical Care	14	0.01
Erb-Downward, John R.	United States	University of Michigan Medical School	37	Microbiology; Microbiome; Lung Microbiome; Small Molecules; Prostaglandins	12	0.01
Cox, Michael J.	Belgium	Janssen Pharmaceutica, Headquarters	25	Respiratory Microbiome; Microbial Ecology; Human Microbiome; Microbiology	11	0.07
Segal, Leopoldo N.	United States	NYU Grossman School of Medicine	30	COPD Inflammation; Lung Microbiome; Immune Response	11	0.02
Lynch, Susan V.	United States	University of California, San Francisco	55	Microbiome	9	0.05
Moffatt, Miriam F.	United Kingdom	National Heart and Lung Institute	75	Respiratory Genetics; Asthma; GWAS; Microbiome	9	0
Morris, Alison	United States	University of Pittsburgh School of Medicine	47	Pulmonary; Allergy; Critical Care Medicine	9	0.02
Sanders, Elisabeth A.M.	Netherlands	Wilhelmina Kinderziekenhuis	63	Pediatric Immunology; Infectious Diseases; Vaccinology	9	0
Wang, Zhang	China	South China Normal University	18	Microbiology; Respiratory System; Experimental Medicine; Biochemistry	9	0
Brightling, Christopher E.	United Kingdom	University of Leicester	113	Respiratory System; Immunology; General and Internal Medicine; Allergy	8	0.06
Chu, Mei Ling J.N.	Netherlands	Rijksinstituut voor Volksgezondheid en Milieu	14	Nuclear Medicine; Medical Imaging; Respiratory System; Immunology	8	0
Curtis, Jeffrey L.	United States	VA Ann Arbor Healthcare System	70	Respiratory System; Immunology; Cell Biology; Biochemistry and Molecular Biology	8	0.03
Cuthbertson, Leah	United Kingdom	National Heart and Lung Institute	18	Respiratory System; Microbiology; Environmental Sciences and Ecology; Medicine	8	0

**Table 3:** Top 20 keywords with the strongest citation bursts.

Keywords	Year	Strength	Begin	End	2003–2023
Diversity	2010	4.03	2010	2017	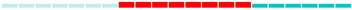
Cystic fibrosis	2010	3.61	2010	2017	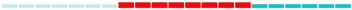
Exacerbations	2011	3.14	2011	2015	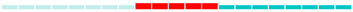
Community	2012	8.1	2012	2017	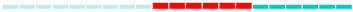
Colonization	2012	4.63	2012	2016	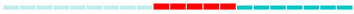
Samples	2012	3.4	2012	2016	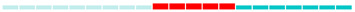
Sputum	2013	6.02	2013	2016	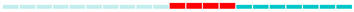
Airway microbiota	2013	3.86	2013	2015	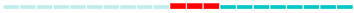
Pneumonia	2013	3.24	2013	2017	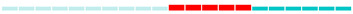
Obstructive pulmonary disease	2011	5.37	2016	2018	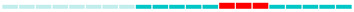
Respiratory tract	2016	4.87	2016	2018	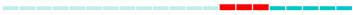
Bacterial infection	2017	3.4	2016	2018	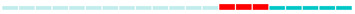
Infants	2016	3.5	2017	2019	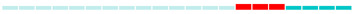
Severity	2017	3.32	2018	2019	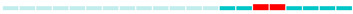
Dynamics	2019	3.27	2018	2020	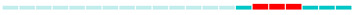
Staphylococcus aureus	2019	3.37	2019	2020	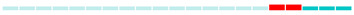
Lung microbiota	2019	4.76	2021	2023	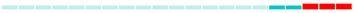
Injury	2021	3.91	2021	2023	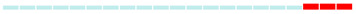
Virus	2018	3.7	2021	2023	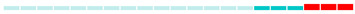
Efficacy	2021	3.13	2021	2023	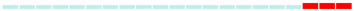

“Year” refers to the initial emergence of the term. “Begin” and “End” indicate the years when the term started and ceased to be a hotspot. In the timeline illustrations, each segment represents a year spanning from 2003 to 2023: light segments indicate the term did not appear during that year, red segments indicate that the term was a hotspot in that year, and dark blue segments show the term’s presence but not as a hotspot in that year.

## Data Availability

The original contributions presented in this study are included in the article/Supplementary Material. Further inquiries can be directed to the corresponding authors.

## Supplementary Materials

The following supporting information can be downloaded at: https://www.mdpi.com/article/10.3390/pathogens13110996/s1, Figure S1: International cooperation network; Figure S2: Journal co-citation network; Figure S3: Paper citation clustering network of Huffnagle, Gary B, Bogaert, Debby, Dickson, Robert P; Figure S4: Keyword co-occurrence clustering; Figure S5: Co-citation network. Figure S6: Double-graph superposition of journal clustering; Table S1: Number of publications by research institutions; Table S2: Top 30 most cited publications.

## Abbreviations

COPD: chronic obstructive pulmonary disease; COVID-19, Coronavirus Disease 2019; H-index, Hirsch index; ICU, intensive care unit; IF, journal impact factor; JCRs, Journal Citation Reports; MeSHs, Medical Subject Headings; WOSCC, Web of Science Core Collection; WOS, Web of Science.
