# Supplementary material for: Bibliometric Analysis of Research Trends and Prospective Directions of Lung Microbiome
Source: Pathogens. 2024 Nov 14;13(11):996. doi: 10.3390/pathogens13110996 (PMC11597221; doi:10.3390/pathogens13110996)
Supplement: Supplementary file 1 [file pathogens-13-00996-s001.zip › pathogens-3272890-supplementary.pdf]

Supplementary Data

Supplementary Table S1. Number of publications by research institutions.

Institution	Number of publications
Imperial Coll London, Natl Heart & Lung Inst, London, England	13
McMaster Univ, Dept Med, Hamilton, ON, Canada	8
Spaarne Gasthuis Acad, Hoofddorp, Netherlands	7
Univ Michigan, Sch Med, Dept Microbiol & Immunol, Ann Arbor, MI 48109 USA	7
Univ Minnesota, Dept Vet & Biomed Sci, St Paul, MN 55108 USA	7
McMaster Univ, Dept Biochem & Biomed Sci, Hamilton, ON, Canada	6
Univ Calgary, Fac Vet Med, Calgary, AB, Canada	6
Icahn Sch Med Mt Sinai, Dept Genet & Genom Sci, New York, NY 10029 USA	5
Spaarne Gasthuis Acad, Haarlem, Netherlands	5
Univ Calgary, Dept Microbiol Immunol & Infect Dis, Calgary, AB, Canada	5
Univ Med Ctr Utrecht, Dept Med Microbiol, Utrecht, Netherlands	5
Univ Michigan, Sch Med, Dept Internal Med, Div Pulm & Crit Care Med, Ann Arbor, MI 48109 USA	5
Univ Pittsburgh, Sch Med, Dept Med, Div Pulm Allergy & Crit Care Med, Pittsburgh, PA 15213 USA	5
Imperial Coll, Natl Heart & Lung Inst, London, England	4
Michigan Ctr Integrat Res Crit Care, Ann Arbor, MI USA	4
Sustainable Sci Inst, Managua, Nicaragua	4
Tsinghua Univ, Sch Med, Beijing, Peoples R China	4
Univ Bern, Inst Infect Dis, Bern, Switzerland	4
Univ Calif San Diego, Dept Med, La Jolla, CA 92093 USA	4
Univ Calif San Francisco, Dept Med, Div Gastroenterol, San Francisco, CA 94143 USA	4
Univ Calif San Francisco, Dept Med, San Francisco, CA USA	4
Univ Lausanne, Lausanne, Switzerland	4
Univ Med Ctr Utrecht, Wilhelmina Childrens Hosp, Dept Paediat Immunol & Infect Dis, Utrecht, Netherlands	4
Univ Michigan, Dept Microbiol & Immunol, Ann Arbor, MI 48109 USA	4
Univ Missouri, Coll Vet Med, Columbia, MO 65211 USA	4
Univ Toronto, Ctr Anal Genome Evolut & Funct, Toronto, ON, Canada	4
Univ Toronto, Dept Cell & Syst Biol, Toronto, ON, Canada	4
Berlin Inst Hlth BIH, Berlin, Germany	3
Guangzhou Med Univ, GMU GIBH Joint Sch Life Sci, Guangzhou, Guangdong, Peoples R China	3
Haukeland Hosp, Dept Thorac Med, Bergen, Norway	3
Hosp Univ Parc Tauli, Dept Microbiol, Sabadell, Spain	3
Icahn Sch Med Mt Sinai, Icahn Inst Genom & Multiscale Biol, Dept Genet & Genom Sci, New York, NY 10029 USA	3
Icahn Sch Med Mt Sinai, Inst Immunol, New York, NY 10029 USA	3
Indiana Univ, Dept Med, Indianapolis, IN USA	3
Lawrence Livermore Natl Lab, Phys & Life Sci Directorate, 7000 East Ave, Livermore, CA 94550 USA	3

McMaster Univ, Dept Pathol & Mol Med, Hamilton, ON, Canada	3
Minist Hlth, Ctr Salud Socrates Flores Vivas, Managua, Nicaragua	3
Nagasaki Univ, Dept Resp Med, Grad Sch Biomed Sci, Nagasaki, Japan	3
Natl Inst Publ Hlth & Environm, Ctr Infect Dis Control, Bilthoven, Netherlands	3
NYU, Sch Med, Dept Med, New York, NY 10016 USA	3
Ohio State Univ, Coll Vet Med, Dept Vet Prevent Med, Columbus, OH 43210 USA	3
Ohio State Univ, Ohio Agr Res & Dev Ctr, Food Anim Hlth Res Program, Wooster, OH 44691 USA	3
Queens Univ Belfast, Sch Med Dent & Biomed Sci, Ctr Infect & Immun, Belfast, Antrim, North Ireland	3
Queens Univ Belfast, Sch Pharm, Belfast, Antrim, North Ireland	3
South China Normal Univ, Sch Life Sci, Inst Ecol Sci, Guangzhou, Guangdong, Peoples R China	3
Southampton Univ Hosp NHS Trust, Cyst Fibrosis Unit, Southampton, Hants, England	3
Spaarne Gasthuis, Dept Obstet & Gynaecol, Hoofddorp, Netherlands	3
Tan Tock Seng Hosp, Dept Resp & Crit Care Med, Singapore, Singapore	3
Univ Arkansas, Dept Anim Sci, Div Agr, Fayetteville, AR 72701 USA	3
Univ Basel, Basel, Switzerland	3
Univ Calgary, Dept Med, Calgary, AB, Canada	3
Univ Calif San Francisco, Dept Anesthesia & Perioperat Care, San Francisco, CA 94143 USA	3
Univ Edinburgh, Roslin Inst, Edinburgh, Midlothian, Scotland	3
Univ Ghent, Lab Pharmaceut Microbiol, Ghent, Belgium	3
Univ Maryland, Sch Med, Inst Genome Sci, Baltimore, MD 21201 USA	3
Univ Michigan, Dept Microbiol & Immunol, Med Sch, Ann Arbor, MI 48109 USA	3
Univ Michigan, Dept Mol Cellular & Dev Biol, Ann Arbor, MI 48109 USA	3
Univ Michigan, Dept Mol Cellular & Dev Biol, Ann Arbor, MI USA	3
Univ Michigan, Sch Publ Hlth, Dept Biostat, Ann Arbor, MI 48109 USA	3
Univ Minnesota, Dept Med, Minneapolis, MN 55455 USA	3
Univ Minnesota, Midcent Res & Outreach Ctr, Willmar, MN USA	3
Univ Missouri, Coll Vet Med, Dept Vet Med & Surg, Columbia, MO 65211 USA	3
Univ Missouri, Metagen Ctr, Columbia, MO 65211 USA	3
Univ Penn, Perelman Sch Med, Dept Microbiol, Philadelphia, PA 19104 USA	3
Univ Pittsburgh, Ctr Med & Microbiome, Pittsburgh, PA 15260 USA	3
Univ Pittsburgh, Dept Computat & Syst Biol, Pittsburgh, PA USA	3
Univ Pittsburgh, Sch Med, Dept Immunol, Pittsburgh, PA USA	3
Univ Pretoria, Dept Internal Med, Pretoria, South Africa	3
Univ Pretoria, Dept Med Microbiol, Pretoria, South Africa	3
Univ Toronto, Dept Lab Med & Pathobiol, Toronto, ON, Canada	3
VA San Diego Healthcare Syst, Res Serv, San Diego, CA 92161 USA	3

Supplementary Table S2. Top 30 most cited publications

Author	Journal	Year	Centrality	Citation Count	DOI
Bolyen E	Nat Biotechnol	2019	0.05	66	10.1038/s41587-019-0209-9
Man WH	Nat Rev Microbiol	2017	0.07	61	10.1038/nrmicro.2017.14
Budden KF	Nat Rev Microbiol	2017	0.17	59	10.1038/nrmicro.2016.142
Dickson RP	Mbio	2017	0.04	54	10.1128/mBio.02287-16
Bassis CM	Mbio	2015	0.17	51	10.1128/mBio.00037-15
Dickson RP	Annu Rev Physiol	2016	0.06	51	10.1146/annurev-physiol-021115-105238
Morris A	Am J Resp Crit Care	2013	0.12	49	10.1164/rccm.201210-1913OC
Callahan BJ	Nat Methods	2016	0.01	47	10.1038/NMETH.3869
Teo SM	Cell Host Microbe	2015	0.04	45	10.1016/j.chom.2015.03.008
Dickson RP	Am J Resp Crit Care	2020	0.03	43	10.1164/rccm.201907-1487OC
Segal LN	Nat Microbiol	2016	0.07	40	10.1038/NMICROBIOL.2016.31
Davis NM	Microbiome	2018	0.08	38	10.1186/s40168-018-0605-2
Erb-Downward JR	Plos One	2011	0.03	37	10.1371/journal.pone.0016384
Jin CC	Cell	2019	0.04	35	10.1016/j.cell.2018.12.040
R Core Team	R Language Env Stat	2023	0.11	34	
Routy B	Science	2018	0.07	33	10.1126/science.aan3706
Dickson RP	Ann Am Thorac Soc	2015	0.05	33	10.1513/AnnalsATS.201501-029OC
Charlson ES	Am J Resp Crit Care	2011	0.01	33	10.1164/rccm.201104-0655OC
Segal LN	Microbiome	2013	0.08	32	10.1186/2049-2618-1-19
Budden KF	Lancet Resp Med	2019	0.03	32	10.1016/S2213-2600(18)30510-1
Salter SJ	Bmc Biol	2014	0.03	32	10.1186/s12915-014-0087-z
Greathouse KL	Genome Biol	2018	0.02	31	10.1186/s13059-018-1501-6
Tsay JCJ	Am J Resp Crit Care	2018	0.06	30	10.1164/rccm.201710-2118OC
Sze MA	Am J Resp Crit Care	2012	0	30	10.1164/rccm.201111-2075OC
Wypych TP	Nat Immunol	2019	0.06	28	10.1038/s41590-019-0451-9
Liu HX	Int J Cancer	2018	0.05	28	10.1002/ijc.31098
Dickson RP	Am J Resp Crit Care	2018	0.02	28	10.1164/rccm.201711-2180OC
Schuijt TJ	Gut	2016	0.05	27	10.1136/gutjnl-2015-309728
Huffnagle GB	Mucosal Immunol	2017	0.04	27	10.1038/mi.2016.108
Dang AT	Mucosal Immunol	2019	0.03	27	10.1038/s41385-019-0160-6

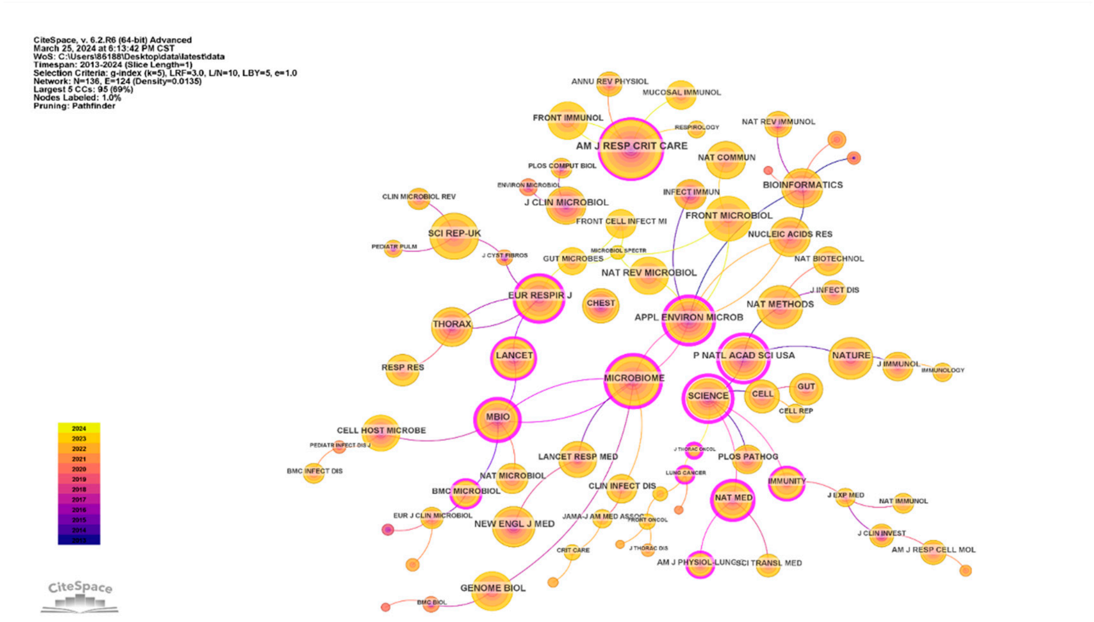

Figure S1. International cooperation network.

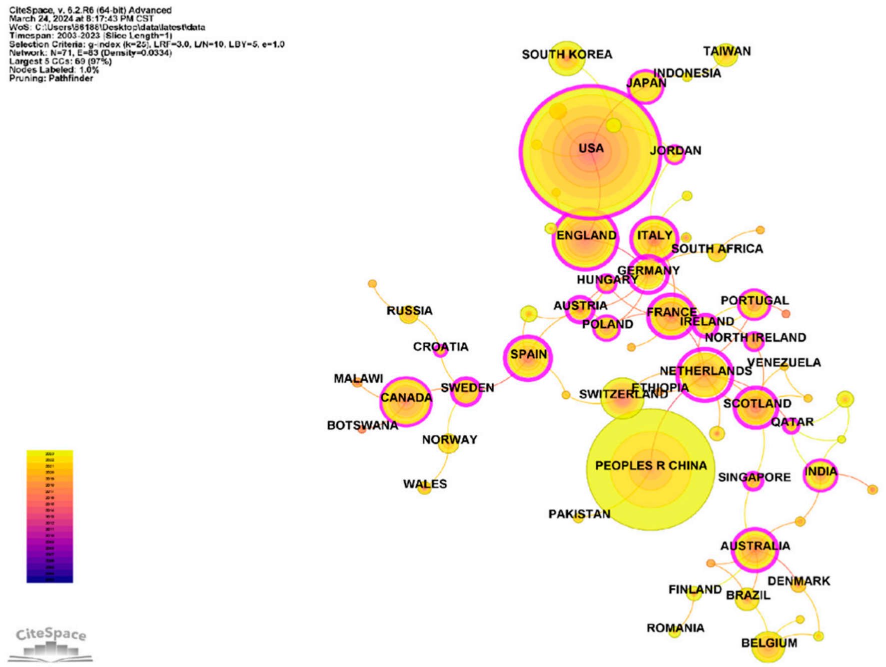

Figure S2. Journal co-citation network.

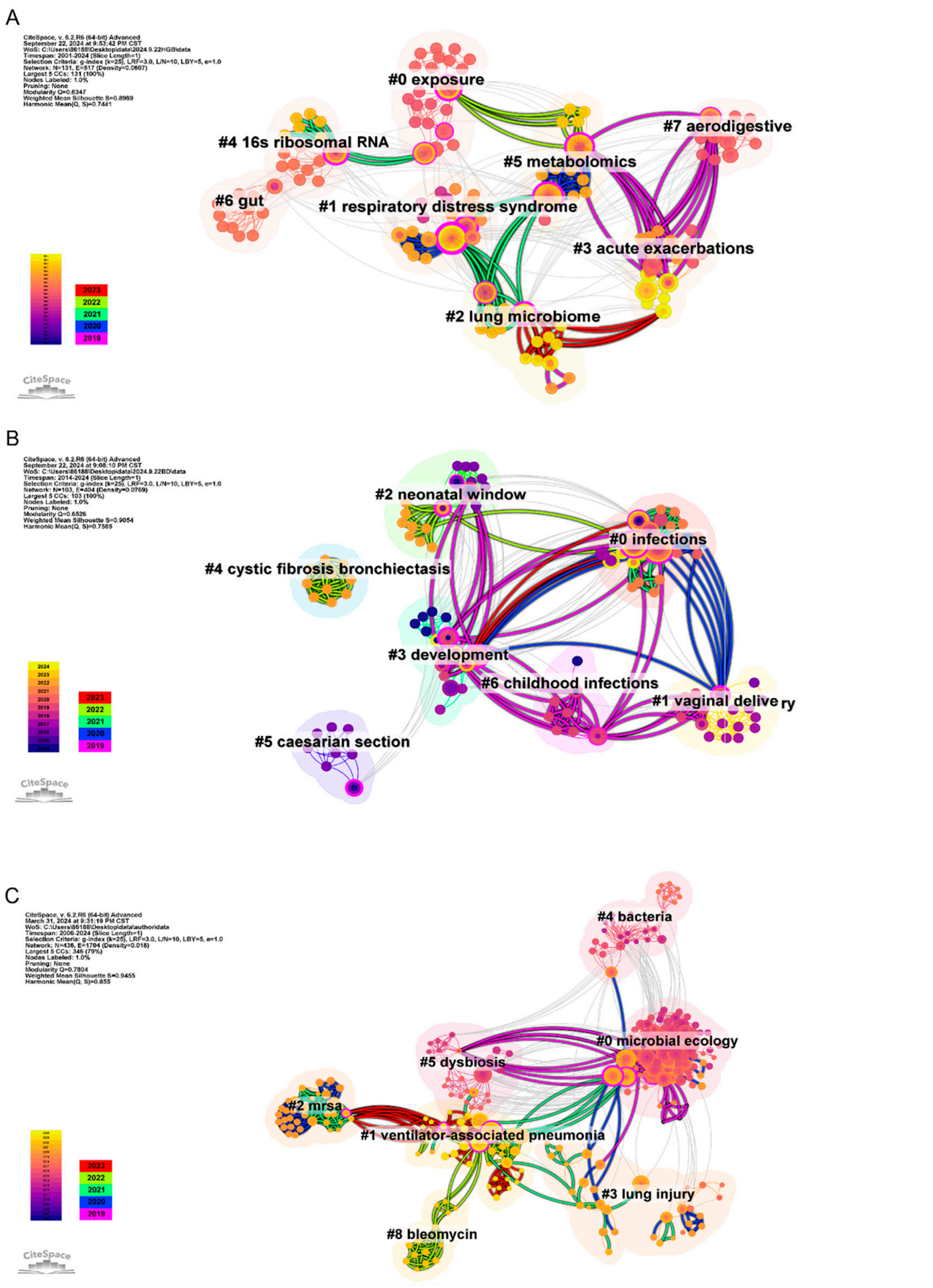

Figure S3. Paper citation clustering network of Huffnagle, Gary B, Bogaert, Debby, Dickson, Robert P.

(A) Paper citation clustering network of Huffnagle, Gary B.

(B) Paper citation clustering network of Bogaert, Debby

(C) Paper citation clustering network of Dickson, Robert P.

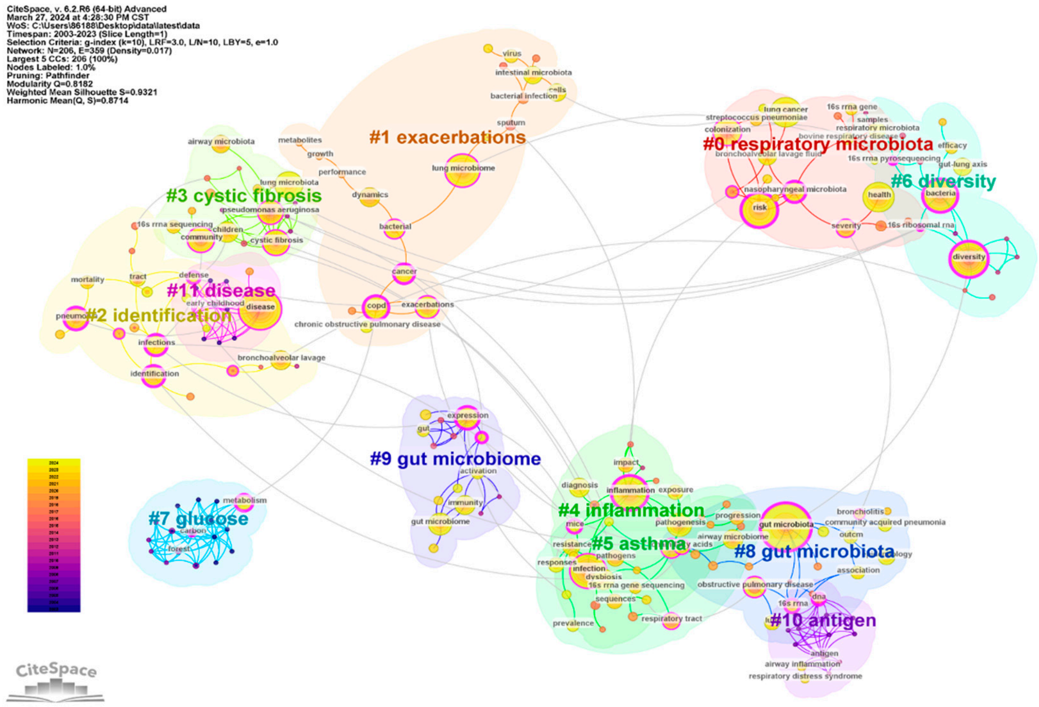

Figure S4. Keyword co-occurrence clustering.

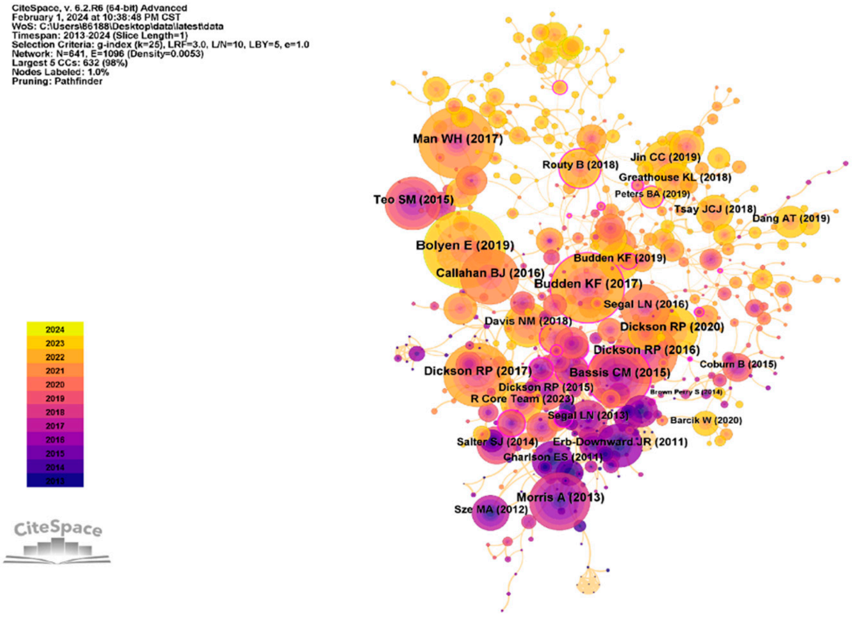

Figure S5. Co-citation network.

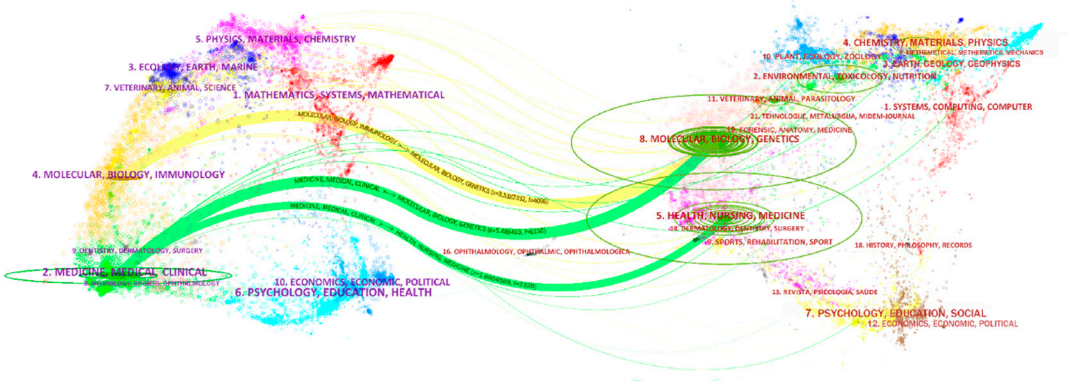

Figure S6. Double-graph superposition of journal clustering.
